# Being Mindful About Overuse of Total Scores: a Comparison of Total Scores and Moderated Nonlinear Factor Analysis Scores in Assessing Mindfulness Across Race/Ethnicity, Age, and PTSD Diagnosis

**DOI:** 10.1007/s11121-025-01862-3

**Published:** 2025-12-09

**Authors:** Alyssa Lozano, Lissette M. Saavedra, Tara G. Bautista, Mariana Sanchez, Antonio A. Morgan-López, Hortensia Amaro

**Affiliations:** 1 School of Nursing and Health Studies, University of Miami, Coral Gables, FL USA; 2 RTI International, Community Health Research Division, Research Triangle Park, Raleigh, NC USA; 3 Department of Health, Exercise, and Sports Science, University of New Mexico, Albuquerque, NM USA; 4 Herbert Wertheim College of Medicine and Robert Stempel College of Public Health and Social Work, Florida International University, Miami, FL USA

**Keywords:** Mindfulness, psychometrics, Hispanic, PTSD, women

## Abstract

Although mindfulness-based interventions show initial positive results on a range of substance use behaviors, evaluations of mindfulness-based interventions would benefit from state-of-the-art alternative approaches to the ubiquitous use of sum or total scores. Sum scores do not reflect “true” underlying mindfulness as they do not consider differences in the relative weight of each item and/or the possibility that measurement may differ across groups. The purpose of this study was to identify measurement noninvariance/differential item functioning (MNI/DIF) across racial and ethnic groups, age groups, and those with PTSD diagnoses and differences in inferences on the factors of the Five Facet Mindfulness Questionnaire between scale scores estimated using moderated nonlinear factor analysis (MNLFA) and a total score analog model (TSA). Age, PTSD diagnosis, non-Hispanic Black race/ethnicity, Hispanic race/ethnicity, and other race/ethnicity showed statistically significant MNI/DIF. In the MNLFA model, PTSD diagnosis and Hispanic race/ethnicity contributed to significant MNI/DIF on the “true” acting with awareness, describing, and observing latent factors such that Hispanic participants were higher on average on acting with awareness scores and lower on average on describing and observing scores. The TSA model failed to estimate significant differences on acting with awareness score for participants with PTSD diagnosis. Additionally, in the TSA model, there was an increase in the effect size of Hispanic participants’ baseline describing and observing estimates, thus overestimating differences in respective scores for Hispanic participants. Failing to correct for MNI/DIF in mindfulness scale scores can impact inferences and effect sizes for group differences in mindfulness thus creating bias in characterizing mindfulness, particularly for Hispanic individuals and those with PTSD diagnoses.

## Introduction

Mindfulness, widely considered to be an inherent quality of human consciousness (Black, 2011), is commonly defined as “paying attention in a particular way: on purpose, in the present moment, and nonjudgmentally” within the Western academic and scientific context (Kabat-Zinn, 2023). Mindfulness can be assessed as current moment awareness, referred to as “state mindfulness” or as a common way of being or characteristic of an individual, referred to as “trait mindfulness.” While trait mindfulness is typically measured as a steady concept, there is some evidence of increases in trait mindfulness following training in mindfulness practices (Kiken et al., 2015). This suggests that mindfulness training through exposure to mindfulness-based interventions (MBIs) may increase trait and state mindfulness, and careful measurement is critical for interventionists.

There is a growing body of evidence that supports the utility of MBIs in preventing and reducing stress-related conditions, including anxiety, depression, and substance use (Bowen & Marlatt, 2009; Bowen et al., 2014; Roemer et al., 2008; Teasdale et al., 2000). There is evidence that MBIs are acceptable and effective among ethnoracially diverse populations (Castellanos et al., 2020), with some studies finding a greater effect of MBIs (as compared to treatment as usual) among racial/ethnic minoritized participants (Vinci, 2020; Webb Hooper et al., 2018; Witkiewitz et al., 2013). Yet, most mindfulness protocols and measurements were created, tested, and validated within non-Hispanic White samples of people (Nagy et al., 2022).

Along with the large body of evidence to support the association between mindfulness and physical and mental health outcomes, there are numerous existing instruments to measure mindfulness orientation at the individual level (Miller et al., 2024). A recent critical review of mindfulness measures identified fourteen tools, published between 2001 and 2021, assessing mindfulness as a state, trait, and process (Miller et al., 2024). Based on five already existing validated mindfulness scales, the Five Facets of Mindfulness Questionnaire (FFMQ) is one of the most widely used reliable and valid measures and has been translated into other languages (Asensio-Martínez et al., 2019; Khanjani et al., 2022). Nevertheless, the need for ongoing research to refine mindfulness measurement tools, ensuring they are valid, reliable, and applicable across different contexts and populations, remains an ongoing issue (Pallozzi et al., 2017).

Prior work has identified differences in how individuals with PTSD report trait mindfulness, including higher scores on acting with awareness that may reflect hypervigilance or avoidance (Price & Hooven, 2018). Age-related variation in mindfulness has also been observed in clinical samples though inconsistently (Shook et al., 2017; Spijkerman et al., 2016) warranting further exploration. Specifically, previous studies have found that older adults score significantly higher on mindfulness compared to their younger counterparts (Johnson & Kil, 2025; Mahlo & and Windsor, 2021). For racially and minoritized individuals, including Hispanic people, growing attention has been paid to the importance of culturally congruent mindfulness measurement (Castellanos et al., 2020; Nagy et al., 2022). These studies underscore the need to investigate whether observed group differences reflect true variation or are artifacts of measurement bias such as differential item functioning (McNeish, 2016; McNeish & Wolf, 2020). Evaluating the impact of mindfulness-based interventions on substance use behaviors would benefit from state-of-the-art alternative approaches to the ubiquitous use of sum or total scores. Several studies have utilized the FFMQ in the evaluation of substance use-related outcomes (Bowen & Enkema, 2014; Cavicchioli et al., 2023; Killeen et al., 2023; Mallik et al., 2021; Marchand et al., 2021; Single & Keough, 2020). However, these studies evaluate either the overall mindfulness total score or the total scores for each of the five subscales. This is concerning given that a growing body of research shows sum scores do not reflect the “true” underlying mindfulness, as these approaches do not consider the possibility that measures of mindfulness (a) have items with different “weights” (as opposed to assuming each item is equally weighted), which is undercut when items are summed (McNeish, 2022; McNeish & Wolf, 2020), and (b) may differ across groups (Morgan-López et al., 2022, 2023). Scale score estimation approaches that consider item/symptom weighting in the scoring of primary outcomes and the extent to which measurement bias/differential item functioning (DIF) is examined across time, populations, is critical. This is particularly true for variables that are commonly used as covariates such as race/ethnicity, age, and PTSD diagnosis; it is critical to disentangle how much of the impact of hypothesized covariates reflect true differences in the underlying construct of interest (in this case, mindfulness) rather than artifacts of the measurement process. For example, item/symptoms from established measures can be scored under factor analysis and item response theory (FA/IRT) using item parameters that are estimated as part of a within-paper supplemental analysis with the sample that is being studied. In contexts with small samples (e.g., < 100), such as those often observed in treatment outcome studies, Bayesian FA/IRT with properly specified prior information would provide a superior option for scale score estimation that, among other advantages, can avoid estimation problems that can occur in small samples under frequentist estimation procedures such as maximum likelihood (MacCallum et al., 1999; McNeish, 2016; Muthén & Asparouhov, 2012). Additionally, prior research conducted with the current sample found a positive association between baseline PTSD symptom severity and uptake of mindfulness practice at the end of the intervention, suggesting the PTSD symptoms may influence the participant engagement with the intervention curriculum (Bautista & Amaro, 2023). Another study using data from this sample found that quantitative scores on satisfaction and qualitative themes related to satisfaction with the intervention at session 2 and session 11 differed based on PTSD symptom severity (Bautista et al., 2024). These findings support the need to investigate PTSD diagnosis as a covariate that reflects true differences in the measurement of mindfulness, rather than artifacts of the measurement process. This is especially important when assessing intervention response with minoritized individuals. There is a need for culturally tailored mindfulness-based approaches among racial-ethnic minoritized populations (Sanchez et al., 2024), but also for accurate and precise measurement that accounts for cultural worldviews, ideologies, and values of various racial and ethnic groups that may create bias in characterizing mindfulness among various racial/ethnic groups. Thus, there is also a need to maximize precision in measurement in addiction science as it relates to (a) screening for equitable prevention and treatment inclusion (both clinical trials and community settings), (b) monitoring of individual-patient outcomes, and (c) characterizing overall treatment effectiveness (Alegría et al., 2018). We have argued and demonstrated the benefits of incorporating recent methodological advances, particularly as reflected in advanced psychometric methodologies, for better understanding the multifaceted influences and mechanisms in individuals who have been underrepresented in research, which requires a shift in our approach to measurement (Sanchez et al., 2024)*.* Towards this aim, the purpose of this study was to identify the following: (1) measurement noninvariance/differential item functioning (MNI/DIF) across racial and ethnic groups, age groups, or those with PTSD diagnoses and (2) differences in inferences regarding measured mindfulness between scale scores estimated using moderated nonlinear factor analysis (MNLFA) and a total score analog model (TSA).

## Method

### Participants and Procedures

Data for the present study came from the baseline interview of a large randomized clinical trial of a mindfulness-based relapse prevention adjunctive intervention program for women (*N* = 245) in residential substance use disorder (SUD) treatment (Amaro & Black, 2017; Black & Amaro, 2019). Participants in the parent study were recruited at entry into a gender-specific residential SUD treatment program. The research interviewer made appointments with prospective participants, conducted the informed consent and HIPAA processes, and administered the baseline assessment. All data used in the present study were collected during the baseline interview. Inclusion criteria were adults between the ages of 18 and 65, SUD diagnosis via clinical record, ability to provide consent (i.e., English fluency, no cognitive impairment), and willingness to be audio recorded and provide broad consent for other studies using the collected data. Exclusion criteria included conditions for which the intervention was contraindicated or that could potentially interfere with the intervention (i.e., untreated psychotic disorder, suicidal ideation in the last 30 days, or other untreated chronic serious mental health disorder), current imprisonment, more than 6 months pregnant, and not willing to sign a HIPAA form or be audio recorded during interviews and intervention sessions. Intervention results of the parent study are provided elsewhere (Amaro & Black, 2021; Black & Amaro, 2019). Data were collected via in-person interviews by a trained research staff using Research Electronic Data Capture. All participants completed informed consent, and all parent study procedures were approved by the University of Southern California Institutional Review Board. Data used in the study are available upon reasonable request.

### Measures

#### Predictors

Race and ethnicity (i.e., Non-Hispanic White, Non-Hispanic Black, Hispanic 1,  and other race and ethnicity), age (measured as a continuous variable), and posttraumatic stress disorder (PTSD) diagnosis were examined as predictors of MNI/DIF and latent underlying mindfulness (see Five Facet Mindfulness Questionnaire below). Dummy variables were created for the race and ethnicity categories with “Non-Hispanic White” race and ethnicity as the reference variable. For PTSD diagnosis, participants were assessed by a clinician within the first 30 days of treatment to ascertain client’s substance use diagnosis and to determine any co-occurring mental health diagnoses.

#### Five Facet Mindfulness Questionnaire

The Five Facet Mindfulness Questionnaire (FFMQ) assesses five facets of general tendencies of being mindful in daily life (Baer et al., 2006). The five facets include the following: observing, the propensity to observe internal and external experiences; describing, describing internal experiences with words; acting with awareness, acting with awareness of the present; nonjudging, taking a nonjudgmental stance toward one’s inner experiences; and nonreactivity, let one’s thoughts and feelings go without focusing or elaborating on them. Sample items asked questions such as the following: “I could easily put my beliefs, opinions, and expectations into words” and “I noticed the smells and aromas of things (or I noticed how things smell).” Response options ranged from 1—never or very rarely true—to 5—very often or always true.

### Data Analytic Plan

We first conducted preliminary tests of model fit. This was conducted using means-and-variance-adjusted weighted least squares (WLSMV) estimation for categorical indicators in M*plus.* We first fit a series of factor models to the data following the structure specified by Pelham III et al. (2019) which fit five separate models for each of the five factors of the FFMQ using IRT. Next, we determined model fit for the psychometric analog(s) to total scores by examining models with equality constraints on the factor loadings for each of the factors (McNeish & Wolf, 2020). Model fit was examined using the Comparative Fit Index (CFI) and Root Mean Square Error of Approximation (RMSEA). “Rules of thumb” have suggested that values >.90 for the CFI and values of <.05 for the RMSEA indicate good fit (Hu & Bentler, 1999; Yu, 2002). Third, a series of moderated non-linear factor analyses models were fit to examine measurement noninvariance/differential item functioning (MNI/DIF) across racial and ethnic groups, age, and those with PTSD diagnoses. We assessed whether each predictor contributed to significant MNI/DIF above and beyond their effects on “true” scores on each of the five factors. In MNLFA, item-level threshold (difficulty) and discrimination parameters are estimated. The threshold parameter reflects the level of the latent trait at which an individual has a 50% probability of endorsing a given response category or higher, while the discrimination parameter reflects how well an item differentiates between individuals with varying levels of the trait (i.e., factor loading).

The MNI/DIF parameters that were significant at *p* ≤.05 were then retained for a global MNLFA model. The parameters that remained significant in the global model were retained for the final MNLFA scale score estimation model to determine the final parameters that were significant and the predictors of the respective factors. We also estimated predictors of the five factors in a total score analog (TSA) model to compare the effect sizes of the estimates. Corresponding code for MNLFA analysis can be found in Bauer (2017) and a tutorial on the approach can be found in Kush et al. (2023).

## Results

### Descriptive Findings

As shown in Table 1, the sample consisted of 245 women in residential treatment for substance use disorder. The average age of participants was 32.2 (SD = 8). The majority identified as Hispanic (57.6%), 20.4% as non-Hispanic White, 20.4% as non-Hispanic Black, and 1.6% as Other. Most participants reported never being married (74.3%), had at least a high school education (52.2%), and in the 8 months prior to treatment entry had used methamphetamines (77.6%), marijuana (55.9%), and alcohol until intoxication (50.6%). Approximately one quarter of participants were diagnosed with post-traumatic stress disorder (PTSD), 16.3% were diagnosed with depressive disorder, and 26.9% had received other mental health diagnoses.

**Table 1:** Sample descriptives (*N* = 245)

Variables	Overall sample (*n* = 245)		PTSD diagnosis	No PTSD diagnosis	Non-Hispanic White people	Non-Hispanic Black people	Hispanic people	Other
**Age, *M (SD)***	32.31 (8.87)		34.00 (8.86)	31.56 (8.81)	34.54 (8.50)***	36.65 (12.14)***	29.63 (6.41)***	38.24 (10.61)***
**Education, *n* (%)**								
< High school	117 (47.8)		32 (27.4)	85 (72.6)	11 (4.5)***	23 (9.4)***	81 (33.1)***	2 (0.8)***
Completed high school	67 (27.3)		16 (23.9)	51 (76.1)	15 (6.1)***	14 (5.7)***	38 (15.5)***	0***
Some education after high school	61 (24.9)		17 (27.9)	44 (72.1)	24 (9.8)***	13 (5.3)***	22 (9.0)***	2 (0.8)***
**Housing status in 8 months prior to treatment, *n* (%)**								
Homeless	56 (22.9)		20 (35.7)	36 (64.3)	10 (4.1)	14 (5.7)	30 (12.2)	2 (0.8)
Unstable accommodations	22 (9.0)		3 (13.6)	19 (86.4)	3 (1.2)	5 (2.0)	13 (5.3)	1 (0.4)
Institution	43 (17.6)		10 (23.3)	33 (76.7)	6 (2.4)	8 (3.3)	29 (11.8)	0
Own place	44 (18.0)		15 (34.1)	29 (65.9)	16 (6.5)	9 (3.7)	19 (7.8)	0
Someone else’s place	80 (32.7)		17 (21.3)	63 (78.8)	15 (6.1)	14 (5.7)	50 (20.4)	1 (0.4)
**Marital status, *n* (%)**								
Married	17 (6.9)		6 (35.3)	11 (64.7)	6 (2.4)	5 (2.0)	6 (2.4)	0
Separated, divorced, or widowed	46 (18.8)		8 (17.4)	38 (82.6)	13 (5.3)	9 (3.7)	23 (9.4)	1 (0.4)
Never married	182 (74.3)		51 (28.0)	131 (72.0)	31 (12.7)	36 (14.7)	112 (45.7)	3 (1.2)
**Top substance used in 8 months prior to treatment entry, *n* (%)**^**a**^								
Methamphetamines	190 (77.6)		49 (25.8)	141 (74.2)	39 (20.5)***	26 (13.7)***	122 (64.2)***	3 (1.6)***
Marijuana	137 (55.9)		36 (26.3)	101 (73.7)	23 (16.8)	26 (19.0)	87 (63.5)	1 (0.7)
Alcohol to intoxication	124 (50.6)		34 (27.4)	90 (72.6)	25 (20.2)	20 (16.1)	75 (60.5)	4 (3.2)
Cocaine	19 (7.8)		5 (26.3)	14 (73.7)	2 (10.5)	7 (36.8)	10 (52.6)	0
Crack	14 (5.7)		7 (50.0)*	7 (50.0)*	0***	12 (85.7)***	2 (14.3)***	0***
**Mental health diagnosis other than SUD, *n* (%)**^**b**^	138 (57.7)							
Post-traumatic stress disorder diagnosis	65 (26.5)		65 (100.0)	0 (00.0)	12 (18.5)	20 (30.8)	33 (50.8)	0
Depressive disorder diagnosis	40 (16.3)		12 (30.3)	28 (70.0)	9 (22.5)	10 (25.0)	21 (52.5)	0
Other mental health diagnosis	66 (26.9)		17 (25.8)	49 (74.2)	23 (34.8)***	18 (27.3)***	24 (36.4)***	1 (1.5)***
**Five Facets Mindfulness Questionnaire*, M (SD)***	76.69 (13.15)		78.20 (12.91)	76.15 (13.23)	79.5 (14.79)	80.2 (11.37)	74.4 (12.60)	78.0 (19.44)
Non-Judge	15.51 (4.28)		15.18 (4.41)	15.63 (4.41)	14.86 (4.17)	15.52 (3.91)	15.78 (4.41)	14.00 (6.16)
Non-Reactivity	11.83 (3.31)		11.78 (3.44)	11.85 (3.27)	11.64 (3.43)	12.42 (3.90)	11.65 (3.06)	13.50 (1.73)
Observe	20.17 (5.23)		19.90 (5.74)	20.27 (5.04)	21.74 (4.35)**	21.24 (4.78)**	19.21 (5.47)**	21.25 (6.13)**
Act with Awareness	23.60 (6.29)		23.11 (6.65)*	24.97 (4.95)*	23.76 (6.92)	25.04 (5.83)	23.10 (6.22)	21.25 (4.19)
**MNLFA Five Facets Mindfulness Questionnaire*, M (SD)***		.02 (.96)	.11 (.98)	−.01 (.96)	.30 (1.05)	.23 (.93)	−.14 (.90)	−.04 (1.55)
Non-Judge	.0009 (.91)		−.10 (.82)	.04 (.96)	−.08 (.81)	.03 (.86)	.02 (.96)	−.01 (.90)
Non-Reactivity	.0011 (.83)		.01 (.89)	−.0017 (.81)	−.03 (.73)	.10 (.98)	−.02 (.82)	.04 (.35)
Observe	.0002 (.86)		−.02 (.94)	.01 (.83)	.08 (.66)	.10 (.92)	−.07 (.90)	.07 (.70)
Act with Awareness	−.0013 (.91)		.14 (.72)	−.05 (.97)	−.07 (.91)	.12 (.92)	−.02 (.92)	−.14 (.39)

Percentages are valid percentages. *p <.05, **p <.01, ****p <.001, ^a^Percentages add up to more than 100% due to use of multiple substances. ^b^Missing data (*n* = 6)

### Preliminary Tests of Model Fit

Preliminary tests of model fit (i.e., CFI and RMSEA) entailed examining five models representing each of the five factors of the FFMQ. The observing facet showed excellent fit (CFI = 1.00, RMSEA =.000, 95% CI [.000,.108]). However, the remaining models showed mixed results. While CFI values ranged from.93 to.98, RMSEA values exceeded conventional thresholds for acceptable fit (describing: CFI =.94, RMSEA =.24, 95% CI [.198,.293]; acting with awareness: CFI =.98, RMSEA =.132, 95% CI [.085,.183]; nonjudging: CFI =.93, RMSEA =.176, 95% CI [.129,.226]; and nonreactivity: CFI =.95, RMSEA =.105, 95% CI [.057,.158]). This may suggest configural differences on some mindfulness subdimensions between the original population on which the FFMQ was developed and this factor structure was based(Pelham III et al., 2019) versus the current sample (Amaro & Black, 2017).

### MNLFA

Item parameters from the final MNLFA model are presented in Tables 2 and 3, with all predictors of MNI/DIF and mindfulness centered so all comparisons are made against the sample means. Notably, almost half of the FFMQ items showed DIF across at least one predictor.

**Table 2:** Final moderated nonlinear factor analysis threshold/difficulty parameter estimates

FFMQ item	Threshold (τ; 0 to 1)	Threshold (1 to 2)	Threshold (2 to 3)	Threshold (3 to 4)	PTSD diagnosis	Hispanic people	Other
**Acting with Awareness**							
I found it difficult to stay focused on (or pay attention to) what was happening in the present moment	− 2.02	− 0.36	1.12	2.30			
It seemed I was “running on automatic” (or feeling out of touch) without much awareness of what I was doing	− 3.59	− 1.89	− 0.25	1.52			
I rushed through activities without being really attentive (or without paying attention) to them	− 3.19	− 1.55	0.26	2.05			− 1.24
I did jobs or tasks without being aware of what I was doing	− 6.04	− 3.23	− 1.11	0.55			
I found myself doing things without paying attention	− 5.35	− 2.07	− 0.30	1.87			
**Describe**							
In the past 30 days, I was good at finding the words to describe my feelings	− 3.95	− 2.22	0.30	2.68			
I could easily put my beliefs, opinions, and expectations into words	− 9.73	− 5.33	1.33	6.21			
It was hard for me to find the words to describe what I was thinking	− 2.26	− 0.26	0.57	1.66			
When I felt something in my body, it was hard for me to find the right words to describe it	− 2.41	− 1.27	− 0.19	1.32	0.71		
Even when I felt terribly upset, I found a way to put my feelings into words	− 2.82	− 1.51	0.23	1.85			
**Non-Judging**							
I told myself that I shouldn’t be feeling the way I’m feeling	− 1.88	− 0.28	1.24	2.17			
In the past 30 days, I made judgments about whether my thoughts were good or bad (or thought about whether a thought was good or bad)	− 1.61	0.27	1.97	2.87			
I told myself I shouldn’t be thinking the way I was thinking	− 2.88	− 0.78	1.52	3.10			
In the past 30 days, I thought some of my emotions were bad or inappropriate and I shouldn’t have felt them	− 2.81	− 1.19	0.44	2.13			
I disapproved of myself when I had illogical ideas (or ideas that don't make any sense)	− 2.80	− 1.31	0.17	1.44			
**Non-Reacting**							
I watched my feelings without getting carried away by them	− 2.19	− 0.53	1.06	2.56			
When I had distressing thoughts or images [ones that bothered me], I didn’t let myself be carried away (or taken over) by them	− 2.92	− 1.316	0.67	2.33		0.73	2.07
In the past 30 days, when I have distressing thoughts or images, I felt calm soon after (or I was able to become calm soon after)	− 2.29	− 0.92	1.09	3.18			
When I had distressing thoughts or images (that bothered me) I just noticed them without reacting	− 2.06	− 0.69	0.99	2.70	− 0.81		
When I had distressing thoughts or images, I just noticed them and let them go	− 2.33	− 0.98	0.68	2.08	− 0.66		
**Observing**							
I paid attention to physical experiences, such as the wind in my hair or sun on my face	− 2.07	− 1.05	0.15	1.85			
I paid attention to sounds, such as clocks ticking, birds chirping, or cars passing	− 2.60	− 1.55	− 0.41	1.44			
I noticed the smells and aromas of things (or I noticed how things smell)	− 3.29	− 2.27	− 0.57	1.20			
I noticed colors, shapes, textures, or patterns of light and shadow in art, pictures or in nature	− 2.96	− 1.74	− 0.25	1.49

**Table 3:** Final moderated nonlinear factor analysis loading/discrimination parameter estimates

FFMQ item	Factor loading	Age	Non-Hispanic Black
**Acting with Awareness**			
I found it difficult to stay focused on (or pay attention to) what was happening in the present moment	2.17		
It seemed I was “running on automatic” (or feeling out of touch) without much awareness of what I was doing	1.80	0.07	
I rushed through activities without being really attentive (or without paying attention) to them	2.77		
I did jobs or tasks without being aware of what I was doing	1.28		
I found myself doing things without paying attention	1.67		
**Describe**			
In the past 30 days, I was good at finding the words to describe my feelings	2.77		
I could easily put my beliefs, opinions, and expectations into words	7.52		
It was hard for me to find the words to describe what I was thinking	− 0.61^a^		
When I felt something in my body, it was hard for me to find the right words to describe it	0.70		
Even when I felt terribly upset, I found a way to put my feelings into words	0.03		
**Nonjudging**			
I told myself that I shouldn’t be feeling the way I’m feeling	0.75		
In the past 30 days, I made judgments about whether my thoughts were good or bad (or thought about whether a thought was good or bad)	1.12		
I told myself I shouldn’t be thinking the way I was thinking	1.69		
In the past 30 days, I thought some of my emotions were bad or inappropriate and I shouldn’t have felt them	1.27		
I disapproved of myself when I had illogical ideas (or ideas that don't make any sense)	0.98		
**Nonreacting**			
I watched my feelings without getting carried away by them	1.16		
When I had distressing thoughts or images [ones that bothered me], I didn’t let myself be carried away (or taken over) by them	1.40	0.03	
In the past 30 days, when I have distressing thoughts or images, I felt calm soon after (or I was able to become calm soon after)	1.91		
When I had distressing thoughts or images (that bothered me) I just noticed them without reacting	0.98		0.91
When I had distressing thoughts or images, I just noticed them and let them go	1.71		
**Observing**			
I paid attention to physical experiences, such as the wind in my hair or sun on my face	1.46		
I paid attention to sounds, such as clocks ticking, birds chirping, or cars passing	1.56		
I noticed the smells and aromas of things (or I noticed how things smell)	1.55		
I noticed colors, shapes, textures, or patterns of light and shadow in art, pictures or in nature	2.08		

^a^This factor loading, although negative, was non-significant

### MNI/DIF

#### Threshold/Difficulty

Threshold/difficulty parameters refer to the level required to reach an item endorsement likelihood of 50%. Key predictors that showed statistically significant MNI/DIF for the threshold/difficulty parameters, compared to the overall sample average thresholds/difficulty parameters, included the following: PTSD diagnosis, Hispanic race/ethnicity, and other race/ethnicity (Table 2). Age and non-Hispanic Black race/ethnicity did not emerge as predictors that showed statistically significant MNI/DIF. The average threshold/difficulty DIF [in standard deviation units; (Steinberg & Thissen, 2006)] across FFMQ items that exceed a Cohen’s *d* value of |0.20| included PTSD diagnosis *d* =.55, Hispanic people *d* =.73, and people with other race/ethnicity *d* = 1.66.

#### Loading/Discrimination

Key predictors that showed statistically significant MNI/DIF included age and non-Hispanic Black race/ethnicity (Table 3). Older participants showed a significantly larger-than-average loading/discrimination parameter for an item in the act with awareness domain [*It seemed I was ‘running on automatic’ (or feeling out of touch) without much awareness of what I was doing*] and for an item in the nonreacting domain [*When I had distressing thoughts or images [ones that bothered me], I didn’t let myself be carried away (or taken over) by them*]. Non-Hispanic Black participants showed significantly larger-than-average loading/discrimination parameter on an item from the nonreacting domain [*When I had distressing thoughts or images (that bothered me) I just noticed them without reacting*].

### Predictors of Latent Mindfulness Factors: MNLFA Scores

After accounting for MNI/DIF, PTSD diagnosis emerged as a significant predictor of higher scores for acting with awareness (*d* =.31, *SE* =.12, *z* = 2.35, *p* <.05). This suggests that those with PTSD diagnosis were higher on average on scores for acting with awareness. *Hispanic* race/ethnicity emerged as a predictor of lower scores for describing (*d* =  −.50, *SE* =.13, *z* =  − 2.94, *p* <.01) and observing (*d* =  −.60, *SE* =.13, *z* =  − 4.10, *p* <.001); suggesting that Hispanics were lower on average on scores for describing and observing, respectively.

### Predictors of Latent Mindfulness Factors: TSA Scores

Unlike the MNLFA scores, the TSA scores did not show significant differences on acting with awareness scores for participants with PTSD diagnosis (*d* =.24, *SE* =.61, *z* = 1.91, *p* =.056). As in the MNLFA scores, Hispanic race/ethnicity emerged as a significant predictor of scores for the describing (*d* =  −.44, *SE* =.78, *z* =  − 2.417, *p* <.05) and observing (*d* =  −.47, *SE* =.57, *z* =  − 3.29, *p* <.01) factors in the TSA scores. However, when using these scores, there was an increase in the effect size of Hispanics’ scores on the describing (*d* =  −.50 vs. *d* =  −.44; 12% increase in effect size) and observing (*d* =  −.60 vs. *d* =  −.47; 21.67% increase in effect size) factors suggesting that the TSA scores would have overestimated the differences in describing and observing scores for Hispanics. In the TSA scores, there were significant differences on acting with awareness for older participants (*d* =  −.02, *SE* =.04, *z* =  − 3.29, *p* <.05); however, this finding was not significant when using MNLFA scores (*d* =  −.01, *SE* =.01, *z* =  − 3.29, *p* =.19).

## Discussion

The purpose of this study was to identify the following: (1) measurement noninvariance/differential item functioning (MNI/DIF) on the widely used Five Facet Mindfulness Questionnaire across racial and ethnic groups, age groups, and those with PTSD diagnoses in women in residential treatment and (2) differences in inferences regarding measured mindfulness between scale scores estimated using moderated nonlinear factor analysis (MNLFA) and total score analog models (TSA). Study results revealed that age, PTSD diagnosis, non-Hispanic Black race/ethnicity, Hispanic race/ethnicity, and other race/ethnicity showed statistically significant MNI/DIF. We also found that there were practical differences for the acting with awareness, describing, and observing subfactors that depended on scoring method. PTSD diagnosis contributed to significant MNI/DIF on MNLFA scores for the acting with awareness factor such that those with PTSD diagnosis had scores that were higher on average. However, there were not significant differences in TSA scores for acting with awareness among those with PTSD diagnosis. TSA scores highlighted significant differences on acting with awareness for older participants that MNLFA scores did not. Hispanic race/ethnicity contributed to significant MNI/DIF on scores for describing and observing such that Hispanic participants were lower on average for describing and observing scores, suggesting that the total score would exaggerate the differences in these scores.

These findings underscore the importance of cultural and contextual considerations in the measurement of different facets of mindfulness. Among Hispanic populations, it is imperative to ensure measures used are culturally congruent and account for the unique nuances of the responses of people who identify as Hispanic. For racially and ethnically minoritized individuals, including Hispanic people, there is growing recognition that mindfulness measures must be culturally congruent and sensitive to cultural values, beliefs, and practices (Castellanos et al., 2020; Nagy et al., 2022). In our study, describing and observing factors were impacted, such that traditional TSA scores would have overestimated describing and observing scores among Hispanics. This discrepancy underscores the importance of adjusting for DIF in these factors to avoid overstating differences in mindfulness facets for Hispanic people, and thus distorting mindfulness levels across different populations. Overestimation of scores can obscure true changes in modifiable mechanisms of change (e.g., observing and describing facets of mindfulness) which can subsequently impact the development and/or refinement of tailored mindfulness-based interventions that align with unique cultural contexts for Hispanics. Further, given the misfit of overall models for some FFMQ factors in this study, compared to the same structure that fit well elsewhere, it is possible that there are questions regarding *configural* invariance (let alone metric/scalar invariance) of the FFMQ, suggesting that mindfulness may not manifest itself in the same way across populations; the current factor structure was tested and found to fit well in largely White samples in the general population (Pelham III et al., 2019) versus the current sample of largely Hispanic women in residential treatment settings (Amaro & Black, 2017). However, this study is not powered to do a proper measurement study for examining and validating alternative factor structures. Future studies should more systematically examine alternate factor structures for the FFMQ across race/ethnicity, gender, and in treatment-seeking populations.

Although item level DIF patterns may appear heterogeneous, these results align with existing theoretical frameworks linking trauma-related attentional processes and cultural models of self-awareness (Vujanovic et al., 2011). Specifically, higher acting with awareness scores among women with PTSD may reflect compensatory attentional control or hypervigilance rather than enhanced mindful presence, consistent with prior research showing that trauma-related hyperarousal can influence self-monitoring and attentional deployment (Hinton & Lewis-Fernández, 2011; Price & Hooven, 2018). Conversely, the lower describing and observing scores among Hispanic participants may reflect cultural variations in the expression of awareness, where mindfulness is often relational and contextually grounded rather than individually introspective (Castellanos et al., 2020; Nagy et al., 2022). This suggests that some observed DIF may be theoretically meaningful, reflecting differences in how mindfulness constructs are operationalized across sociocultural and trauma contexts rather than random measurement noise. Future research with larger and more diverse samples should test whether these patterns replicate and to what extent they represent true cultural or clinical variation in mindfulness-related processes. Replication in other clinical samples may produce similar findings, as this higher-acuity sample could reflect what providers prioritize in real-world settings.

Presence of impairing traumatic reactions (PTSD) impacted measurement of acting with awareness. Individuals with PTSD had higher scores relative to individuals who did not report PTSD in their acting with awareness ratings. However, significant differences were observed on the acting with awareness subfactor MNLFA scores for people with a PTSD diagnosis; however, these differences were not significant when using the TSA scores. Without accounting for these differences in measurement and scale score estimates, traditional scoring models would have missed differences in acting with awareness among those with PTSD. Individuals with PTSD may engage with mindfulness practices, particularly the facet of acting with awareness, differently due to symptoms like hypervigilance, avoidance, or dissociation. If these nuances are not captured, the traditional scoring approaches may fail to detect meaningful variations or changes in mindfulness related to PTSD, potentially leading to inaccurate conclusions about program efficacy or individual differences.

### Implications

The results of this study shed light on the importance of assessing measurement noninvariance/differential item functioning. This information represents important initial steps to inform future assessment of treatments that incorporate mindfulness among ethnoracially diverse samples and samples with a clinical diagnosis of PTSD. The findings have important implications for initial assessments of mindfulness as the presence of PTSD impacts scores on the acting with awareness factor, whereas traditional sum scores (i.e., TSA models) would have missed this difference. It is not clear if the presence of PTSD has an impact on intervention response among women in residential treatments. Nevertheless, this measurement approach is ideal for capturing these specific impacts. Similarly, considerations of the ways in which culture and race/ethnicity may influence the experience and reports of mindfulness as well as responses during the assessment process are imperative (Chapman et al., 2018; Hinton & Lewis‐Fernández, 2011). Precise measurement of mindfulness among those with PTSD and Hispanic populations is therefore critical for evaluating the true effects of mindfulness-based programs and ensuring their relevance and impact in future research. Inaccurate measurement of mindfulness among racially and ethnically minoritized groups and those with PTSD may contribute to the frequently reported finding that mindfulness-based interventions are more effective for racially and ethnically minoritized populations and those with PTSD than for non-Hispanic White individuals and individuals who do not have PTSD (Nagy et al., 2022; Price & Hooven, 2018). However, this pattern may reflect measurement bias rather than true differences in intervention response. This has critical implications for interpreting prevention and intervention research findings. To ensure equity and accuracy in program evaluation, constructs hypothesized as mechanisms of change must be re-examined from the ground up, including their conceptualization and measurement, to ensure they validly reflect the experiences of diverse populations.

### Strengths and Limitations

The results of this study should be evaluated in the context of the strengths and limitations. First, the relatively small sample size can restrict the generalizability of the results, as it might not represent the entire population accurately. Moreover, the “other race/ethnicity” group only had four individuals; however, we were still able to detect statistically significant MNI/DIF for threshold/difficulty parameters. While combining all Hispanic participants together in one group is not ideal, we were limited by sample size and statistical power in our ability to disaggregate Hispanic subgroups. Additionally, it is not feasible for clinical trials to have adequate representation from South American, Central American, Mexican, and Caribbean participants in a manner that would allow for meaningful comparisons. Nevertheless, Hispanic populations are culturally, linguistically, and historically diverse, and these differences may influence both the expression and measurement of mindfulness. Future epidemiological studies with greater subgroup representation are needed to identify the heterogeneity of mindfulness-related constructs and scale functioning across Hispanic subgroups. Additionally, relying on interviews as a data collection method can introduce potential biases, such as recall bias and social desirability bias. The study used the Five Facet Mindfulness Questionnaire which is a well-validated and widely used scale. The limitations of recall and social desirability biases can also be seen as strengths, as they prompt researchers to implement rigorous data collection techniques, such as using multiple interviewers or employing standardized questionnaires, to minimize these biases. Examination of the measurement properties in a sample of women in residential treatment with a range of prevalent mental health diagnoses addresses an important gap in the field. Focus on this specific population allowed for a more nuanced examination which can inform targeted interventions and support services for these individuals. However, it remains unclear if the present study findings generalize to men or others who do not identify as women. Extending and replicating the present work is important to determine whether these findings are unique to women in these settings. Also, as with any study examining racial/ethnic differences, the need to have adequate sample size and proper representation of diverse groups is important.

## Conclusion

Accurately measuring mindfulness within MBIs is an important and challenging methodological task. The conceptual and operational definitions of mindfulness need to be carefully and thoughtfully considered at the start of MBI studies to be congruent with the population and aim of the intervention. Emerging options offer interventionists options to better capture ways in which race/ethnicity, but also specific mental health conditions may impact baseline assessment but also open up opportunities to examine more nuanced treatment responses. Efforts to incorporate these approaches into standard assessment and outcome measurement are already showing promise in other addiction spaces (Saavedra et al., 2022). As leaders in the field continue to call for feasible equity-centered assessment processes approaches (Alegría et al., 2006; Jordan et al., 2020), we offer accessible options to better maximize precision in characterizing screening for equitable treatment inclusion and monitoring of individual patient outcomes and ultimately treatment outcomes. Given the identification of a revised factor structure of the FFMQ in our sample, the current findings provide a necessary foundation for future analysis examining treatment effects and potential racial/ethnic differences in intervention outcomes using MNLFA-based scores. This step is critical for advancing equity in MBIs as failure to account for measurement bias is known to obscure or distort true effects across diverse populations (Sanchez et al., 2024). Accurate measurement is not only a methodological concern but a critical priority for prevention research.

## Footnotes

1 Consistent with US census categories utilized at the time of the study, we use the term Hispanic to represent how the population and this sample identified themselves.
